# Mindfulness-based pain management for patients with chronic musculoskeletal pain: a randomized controlled trial

**DOI:** 10.3389/fmed.2026.1911807

**Published:** 2026-09-28

**Authors:** Holger Cramer, Sanja Krstić, Marleen Schröter, Alina Schleinzer, Hazal Sarak Kucukosmanoglu, Rahele Tavakoly, Petra Meibert, Jörg Meibert, Dennis Anheyer, Heidemarie Haller

**Affiliations:** 1 Institute of General Practice and Interprofessional Care, University Hospital Tübingen, Tübingen, Germany; 2 Robert Bosch Center for Integrative Medicine and Health, Bosch Health Campus, Stuttgart, Germany; 3 Charité – Universitätsmedizin Berlin, Corporate Member of Freie Universität Berlin and Humboldt Universität zu Berlin, Charité Competence Center for Traditional and Integrative Medicine (CCCTIM), Berlin, Germany; 4 Mindfulness Institute Ruhr, Essen, Germany; 5 Department of Psychology and Psychotherapy, Faculty of Health, Witten/Herdecke University, Witten, Germany; 6 Center for Integrative Medicine and Planetary Health, University Hospital Essen, University of Duisburg-Essen, Essen, Germany

**Keywords:** chronic pain, lifestyle & behaviour, MBPM, mindfulness, randomized controlled trial

## Abstract

**Introduction:**

Chronic pain is a significant global public health challenge. There is substantial evidence supporting the benefits of mindfulness-based interventions for chronic pain. Mindfulness-Based Pain Management (MBPM) is an 8-week mindfulness-based behavioral intervention program designed to help individuals manage chronic pain. However, the available evidence regarding the effects of the MBPM program in patients with chronic musculoskeletal pain is limited.

**Materials and methods:**

Adults with chronic musculoskeletal pain were randomly assigned to either the MBPM program group or the waiting-list control group. The MBPM program group received 150 min sessions once a week for eight weeks in addition to usual care. The waiting-list control group only received usual care. The primary outcome was pain acceptance, measured using the Chronic Pain Acceptance Questionnaire (CPAQ) at week 8. Secondary outcomes included pain acceptance at week 26, as well as pain intensity, pain-related disability, health-related quality of life, psychological well-being, pain self-efficacy, mindfulness, self-compassion, and adverse events at weeks 8 and 26.

**Results:**

Forty participants were randomly assigned to one of two groups: the MBPM program group (*n* = 20) or the waiting-list control group (*n* = 20). For the primary outcome, the MBPM program group had a more favorable CPAQ total score at week 8 than the control group (95% confidence interval (CI): −0.4 to 16.3; mean difference (MD) = 7.9; *p* = 0.063). However, this difference was not statistically significant. Among the secondary outcomes, the MBPM program group had higher self-compassion scores at week 8 (MD = 0.3; 95%-CI: 0.0 to 0.6; *p* = 0.035), and higher mindful awareness score at week 26 (MD = 0.3; 95% CI: 0.04 to 0.66; *p* = 0.028) than the control group. No statistically significant differences between the two groups were observed for the other secondary outcomes. No intervention-related adverse events were reported.

**Conclusion:**

In this randomized controlled trial, the MBPM program did not demonstrate a statistically significant effect on pain acceptance compared with the waiting-list control. Nevertheless, between-group comparisons favored MBPM for self-compassion at week 8, and mindful awareness at week 26. However, these findings require confirmation and should be interpreted exploratory. Larger and methodologically rigorous trials with longer follow-up periods are warranted to further evaluate the effectiveness of MBPM for individuals with chronic pain.

**Clinical Trial Registration:** ClinicalTrials.gov, identifier NCT05199805.

## Introduction

Chronic pain is a significant global public health concern that results in a decline in quality of life (1). The actual International Classification of Diseases (ICD-11) classifies chronic pain into seven categories. These include chronic primary pain, such as fibromyalgia and nonspecific low back pain, as well as six types of chronic secondary pain related to underlying conditions, such as musculoskeletal pain. This classification system recognizes chronic pain as a distinct health condition that has both biological and psychosocial dimensions (2). Recent estimates of point prevalence rates range from 20% in developed countries (3, 4) to 33% in low- and middle-income countries (5). In Germany, approximately 20% patients in general medical practices suffer from chronic pain, often in multiple body regions (6). Back pain and other pain conditions have been shown to result in greater functional limitations, an inability to work, and social restrictions (7), often leading to psychological strain (1).

In cases of chronic pain, severe structural pathologies explain the intensity of the pain. Thereby, the resulting impairments are often either absent (8) or not associated with the painful area (9). Instead, psychological, social, and motivational factors have been shown to exert a substantial influence on pain perception and its persistence (8). Catastrophizing and a lack of acceptance of chronic pain have been identified as important factors in pain avoidance and persistence. Conversely, accepting of pain has been shown to facilitate a greater engagement in positive activities and the pursuit of personal goals despite pain. This may result in a reduction in pain-related functional limitations (10). Therefore, both national (8) and international (11, 12) guidelines prioritize nonpharmacological approaches to chronic pain, including conventional and complementary movement and relaxation therapies, psychotherapy, psychosocial interventions, and stress management techniques.

Mindfulness-Based Interventions (MBIs) are a wide array of structured programs, including Mindfulness-Based Stress Reduction (MBSR) and Mindfulness-Based Pain Management (MBPM), which have been adapted for various health conditions (13). MBSR includes mindfulness exercises, gentle physical exercises, and body and breathing awareness practices. These practices are designed to teach individuals how to process thoughts, emotions, and sensations for stress management (14). Accordingly, MBSR programs have demonstrated beneficial effects in patients with chronic pain and are recommended in several international clinical guidelines for managing chronic low back pain, including those of the World Health Organization (WHO) and the American College of Physicians (15, 16). The MBPM program, which adapts the core concepts and practices of MBSR, provides specific applications for individuals living with chronic pain and illness. MBPM incorporates a distinctive emphasis on the following five distinctive components: (1) mindful activity management and pacing, (2) gentle, pain-adapted movements, (3) a progressive approach to mindfulness of painful sensations, (4) an emphasis on self-compassion toward the painful body, and (5) promoting social connectedness to reduce isolation (17). Vidyamala Burch developed the MBPM program (18, 19), which is currently delivered through Breathworks programs (20). MBPM can be situated within the context of second-generation mindfulness protocols. However, its conceptual roots of this program can be traced back to Buddhist and Indian contemplative traditions (21). Overall, MBPM is a specialized adaptation designed for individuals with chronic pain. It integrates mindfulness with pain-specific coping strategies, including pain regulation and acceptance, as well as compassion-based practices (17).

Although MBIs, and specifically MBSR programs, have been widely studied, evidence regarding the specific effects of the MBPM program in patients with chronic musculoskeletal pain remains limited. The first clinical study to evaluate the official Breathworks MBPM program for chronic pain was conducted by Vidyamala Burch's team. The study showed improvements in mindfulness, psychological well-being, and pain acceptance. However, they used an uncontrolled pre-post design (22). A quasi-experimental study found similar results (23). In a clinical trial conducted by Pérez-Fernández et al., patients who received the MBPM program demonstrated moderate to large between-group effect sizes compared with a waiting-list control group receiving usual care across several outcomes. These outcomes included improved pain management and pain acceptance as well as improved mental health-related quality of life and resilience. Conversely, there was reduced analgesic use and lower levels of psychopathological symptoms and negative thoughts. However, participants were allocated according to the order of arrival (21), which introduces a high risk of selection bias due to the lack of random allocation. A recent systematic review also showed that the effects of the MBPM program on patients with chronic musculoskeletal pain have not yet been examined in an randomized controlled trial (RCT) using standard randomization (24).

Therefore, the main aim of this study was to investigate the short-term effectiveness of the structured MBPM program, delivered in addition to usual care, compared to usual care alone for pain acceptance in patients with chronic musculoskeletal pain. Thereby, this study employed a single-blind design with non-stratified block randomization to minimize the likelihood of allocation and analysis bias.

## Materials and methods

### Design

This monocentric, single-blind, RCT with two study arms was conducted in Germany from January to November 2022. The study was reported in accordance with the CONSORT guidelines (25). The Ethics approval was granted by the Ethics Committee of the University of Essen and the trial was registered at ClinicalTrials.gov on January 5th, 2022 (NCT05199805), before study recruitment. The study took place at the Department of Internal and Integrative Medicine at Evangelische Kliniken Essen-Mitte. The intervention was delivered at the Mindfulness Institute Ruhr in Essen (https://www.achtsamkeitsinstitut-ruhr.de), and the study adhered to the Declaration of Helsinki. There was no patient or public involvement in the design, conduct, or reporting of the trial.

### Eligibility criteria

Participants had to be at least 18 years old and have had a chronic specific or nonspecific musculoskeletal pain diagnosis for at least three months to be included in the study. Prior to inclusion, potentially eligible participants had to complete diagnostic procedures and receive a physician-confirmed diagnosis. Participants with stable chronic musculoskeletal disorders were included in the study. These disorders included osteoarthritis (e.g., osteoarthritis of the hip, knee, ankle, shoulder, or hand), chronic low back or neck pain, chronic lumbar or cervical disc disorders, spinal stenosis, rotator cuff lesions, and fibromyalgia.

Participants could not be included in the study if their chronic pain was caused entirely by one of the following conditions, or if they had experienced an acute case of one of these conditions within the last three months: degenerative conditions (e.g., intervertebral disc herniation, scoliosis, or spinal stenosis); neurological diseases (e.g., neuropathies, or multiple sclerosis); spinal injuries (e.g., whiplash, vertebral fractures, or the result of surgery); or tumors in the spinal area. However, osteoarthritis, degenerative diseases, and spinal injuries were not considered as exclusion criteria unless they were newly diagnosed or acutely symptomatic within the last three months. Participants were also excluded if they met any of the following criteria: severe comorbid mental illness (e.g., addiction diagnosis, severe depression), severe comorbid physical illness (e.g., cancer diagnosis within the last five years, severe cardiovascular disease, organ insufficiency (kidney, liver, etc.), acute febrile infection, or other severe neurological illnesses such as epilepsy), pregnancy, ongoing retirement application (e.g., disability, occupational disability, reduced earning capacity, or severe disability), or simultaneous participation in other clinical studies.

### Recruitment

Potential participants were recruited through study announcements on the website of the Department of Internal and Integrative Medicine at Evangelische Kliniken Essen-Mitte in Germany, as well as through press advertisements. Former patients who had received treatment for chronic musculoskeletal pain at the department and had previously agreed to receive updates about ongoing studies were also contacted in writing about the start of a new study. Those who expressed interest in the study received written information about the study to read at their convenience. They were then screened by telephone call to determine their eligibility. The study physician checked the inclusion and exclusion criteria on site by means of a detailed general medical history and an orienting clinical-neurological examination. Potential participants were required to bring their medical reports and examination findings. If “red flags” appeared during the medical examination for inclusion, the patient was advised to undergo further imaging or laboratory examinations and/or seek specialist treatment, depending on the suspected diagnosis and urgency, and could not participate in the study. Participants who were potentially eligible were invited to the department for further formal and medical examinations. All participants were informed about the study and examined by a study physician, and given the opportunity to ask questions and clarify any uncertainties. Additionally, they were required to provide written informed consent to enroll in the study. After enrollment, they were asked to complete the baseline questionnaire. All participants were clearly informed of their right to leave the study at any time, for any reason, with no negative consequences for their treatment.

### Randomization and blinding

Eligible participants who provided written informed consent and completed the baseline measurements (at week 0) were assigned to one of the two study arms using a non-stratified block randomization with randomly varying block lengths. The randomization list was password-protected, and only the biometrician had access to it. Based on this list, the biometrician prepared sealed, sequentially numbered, opaque envelopes containing the intervention assignments. The study physician opened the envelope with the lowest number to reveal the patient's assignment. Due to the nature of the intervention, participants and therapists could not be blinded. However, the biometrician, the data collectors (the study staff responsible for transcribing and verifying written questionnaires into Excel), and data analysts were blinded to the study groups.

### Intervention: MBPM program

Participants in the intervention group received the MBPM program in addition to their usual care. The MBPM program was based on the method of Vidyamala Burch (18, 19). This structured program included mindfulness exercises, meditation, a series of gentle physical exercises, body and breathing awareness practices, mindful pain management, and practical pain regulation methods. Furthermore, the practices of self-compassion and compassion for others were particularly emphasized, and participants were also trained to cultivate mindfulness in everyday situations. The six steps of the eight-week program were titled as follows: 1) Mindfulness Is Possible, 2) Approaching the Unpleasant, 3) Seeking and Finding the Pleasant, 4) Gaining an Expanded Perspective, 5) Connection with Others, and 6) Having a Choice.

The MBPM program consisted of eight weekly in-person sessions, each of which lasted 150 min. Each 150 min session was structured into three main components:

1. Psychoeducational content (approximately 60 min)
2. Meditation practice (approximately 30 min)
3. Group discussion, experience sharing, review of the previous week, and explanation of upcoming homework (approximately 60 min)

The key component is the psychoeducational component. It was fostering awareness of automatic behavioral patterns and contrasting them with mindful, meditative awareness. The psychoeducational component of the intervention did not focus on providing explicit medical information about chronic pain. Instead, it emphasized identifying and modifying of maladaptive automatic response patterns and discussing new behavioral strategies. Core topics included:

- Fight-or-flight responses within the framework of stress theory
- Reduced self-compassion, based on Paul Gilbert' model
- Repression and avoidance vs. open, accepting awareness
- Activity pacing in relation to individual performance capacity
- Effective communication strategies in daily life

Differentiation between pain and the individual's reaction to pain, highlighting the possibility of choosing behavioral responses. Each weekly in-person session also included 30 min of guided meditation practice, typically consisting of two audio exercises, one at the beginning and one at the end. Participants also discussed their meditation experiences for about 60 min, exploring how these related to their daily lives and coping with chronic pain. Additionally, they reviewed the previous week and received an explanation of the upcoming homework. Therefore, participants were provided with a structured course folder containing all instructional materials that explained the theoretical foundations and described the exercises to facilitate independent practice at home. In addition to the written materials, participants received seven downloadable, guided audio practices, each lasting approximately 12–15 min. Participants were encouraged to continue practicing between weekly meetings. However, the exact frequency of practice was not formally monitored.

The MBPM program was conducted by a qualified social pedagogue and a psychologist. Both were trained in MBPM. Both were also mind-body-instructors with years of experience working with patients suffering from chronic pain.

### Control: waiting-list receiving usual care

The control group continued to receive their usual treatment. Changes to their usual care were not monitored during the study period. To ensure ethical compliance, six months after randomization and when the data collection was complete (week 26), the control group was offered an identical program to the intervention group. There were no restrictions regarding permitted adjunct therapies for either group.

### Measurement outcomes and time points

The study had three defined measurement time points:

1. Baseline measurements were taken prior to randomization (week 0). During this phase, data were collected on sociodemographic information (e.g., age, height, gender, weight, and socioeconomic status), as well as on lifestyle and disease-related factors (e.g., activity level, duration of active relaxation, pain duration, previous or current treatments for pain, and therapy expectation).
2. Post-intervention measurements were taken immediately after the intervention (week 8).
3. Follow-up measurements were taken six months after randomization (week 26).

The following outcomes were measured using standardized self-reported questionnaires at the indicated time points.

Primary outcome:

- Total score of pain acceptance at week 8: The German version of the Chronic Pain Acceptance Questionnaire (CPAQ) was used to measure total pain acceptance at week 8 (26). The CPAQ contains 20 items that are rated on a 7-point Likert scale ranging from 0 (“never true”) to 6 (“always true”). This results in a total score ranging from 0 to 120, calculated by summing the scores across all items, with higher scores indicating greater pain acceptance.

Secondary outcomes:

- **Subscale scores of pain acceptance at week 8:** The CPAQ is divided into two subscales: One is the CPAQ-Activity Engagement (CPAQ-AE) subscale (11 items; total sum score range: 0–66), which measures the ability to perform daily tasks and leisure activities despite pain. The other subscale is the CPAQ-Pain Willingness (CPAQ-PW) subscale (9 items; total sum score range: 0–54), which measures the tendency to want to avoid or control pain. Higher total scores on both subscales indicate better outcomes.
- **Pain acceptance at week 26:** The total score and subscales of pain acceptance at week 26 were measured using the CPAQ.
- **Health-related quality of life at weeks 8 and 26:** The Short Form-12 (SF-12), a validated health-related quality of life assessment tool, was used to evaluate health-related quality of life. The SF-12 provides two summary scores: one for physical health and one for mental health (27). Scores range from 0 to 100 for both subscales, with higher scores indicating a better health-related quality of life (28). The subscales scores were calculated using the SPSS syntax provided by Bullinger and Kirchberger in 1998 (29).
- **Functional disability at weeks 8 and 26:** The German version of the 7-item Pain Disability Index (PDI) was used to measure the subjective degree of disability caused by pain problems in daily life activities (30). Scores range from 0 to 10, with higher scores indicate higher disability related to pain. The total score was calculated by summing the scores across all items.
- **Psychological symptoms at weeks 8 and 26:** The German version of the Hospital Anxiety and Depression Scale (HADS) was used to evaluate the extent of anxious and depressive symptoms experienced by patients in the past week. The scale consists of two subscales, one for anxiety (HADS-A), and one for depression (HADS-D). Each subscale has seven items, and was rated from 0 to 3. The total score for each subscale was calculated by adding up the scores of its items. Higher scores indicate higher levels of anxiety or depression (31).
- **Self-efficacy at weeks 8 and 26:** The German version of the Arthritis Self-Efficacy Scale (ASES-D) was used to measure subjective self-efficacy regarding pain and functionality (32). This is an 8-item questionnaire. In the original version, Item 7 asked “How confident are you, compared to other fibromyalgia/rheumatoid patients, that you can somehow manage pain in your daily activities?”. In our version, the term “fibromyalgia/rheumatoid patients” was replaced with “chronic pain patients”. Items were rated on a scale from 1 to 10, with higher scores indicating greater perceived self-efficacy in managing pain and related symptoms. An overall mean score was calculated across all items.
- **Mindfulness at weeks 8 and 26:** The Conscious Presence and Self Control (CPSC) Scale (33), a validated 10-items scale, was used to measure conscious presence and situational awareness. Total scores range from 0 to 3, with higher scores indicating greater mindfulness or situational awareness. An overall mean score was calculated.
- **Self-compassion at weeks 8 and 26:** The German-validated 12-item Self-Compassion Scale (SCS) was used to measure the positive and negative aspects of self-compassion across six subscales: self-kindness vs. self-judgment, common humanity vs. isolation, and mindfulness vs. over-identification. Items are rated on a 1–5 Likert scale, with higher scores indicating greater levels of self-compassion. An overall mean score was calculated.
- **Pain intensity at weeks 8 and 26:** Average pain intensity over the last week was measured using a 3-item Numerical Rating Scale (NRS), which ranges from 0 to 10 points. A score of 0 indicates no pain, and a score of 10 indicates the worst possible pain (34). The mean of all three NRS items was calculated.
- **Therapy expectation at week 0:** At baseline, participants' expectations regarding the effects of the study intervention and their anticipated use of the relaxation exercises were assessed at baseline using the two expectations-items from the Treatment Credibility Scale (TCS) on a 0-to-10 rating scale. Higher scores on this scale indicate greater expectations (35).
- **Therapy rationale at week 8:** The three credibility-items of the TCS were used to evaluate the perceived credibility and acceptability of the intervention after its completion at week 8 and 26 (35).
- **Optional participants' feedback at week 8 and week 26:** To obtain further insights into participants' perceptions and experiences of the MBPM program, qualitative feedback was gathered through free-text sections in the questionnaires.

### Adverse effects at weeks 8 and 26

Adverse effects and intervention-related increases in pain, occurring during and after the treatment, were reported in free-text format. Additionally, participants were asked about any side effects or increased pain experienced during or after the treatment.

### Sample size estimation

The sample size calculation was based on the study by Cusens et al. (22), which reported a large effect size of Cohen's d = 1.04 for pain acceptance when comparing the MBPM program in addition to standard therapy with standard therapy alone. This study was the only available study on pain treatment with the MBPM program, which differs from other MBIs conceptually and technically. Assuming a power (1-*β*) of 80%, a significance level (*α*) of 5%, and an anticipated dropout rate of up to 20%, a two-sided t-test would require a total sample size of 40 patients (20 per group) to detect such an effect.

### Data management and analysis

Data were pseudonymously collected via paper-and-pencil questionnaires that contained an automatically generated number, but no names. This allowed the baseline and follow-up questionnaires to be matched without identifying individuals. The study physician transferred the data to Excel, and another researcher checked it.

A copy blinded to study-arm allocation was provided to the statistician who conducted the analysis. Throughout the trial, the statistician was not given with any information that could help identify the two study groups. Descriptive statistics were computed for the overall population and for the two groups. Descriptive statistics are presented as the mean ± standard deviation (SD), and 95% confidence interval (CI) for continuous data, and as absolute numbers and relative frequencies (percentages) for categorical variables. All analyses were conducted using an intention-to-treat approach, meaning that all randomized participants were analyzed in their original groups, regardless of their adherence to or receipt of the allocated intervention.

Missing data were handled using multiple imputation with the missForest package in R. R uses a nonparametric random forest algorithm to estimate missing values. This approach is particularly suitable for datasets containing both continuous and categorical variables because it imputes missing data iteratively based on predictive modeling until convergence is achieved.

The primary outcome measure was analyzed using a univariate analysis of covariance (ANCOVA) as the confirmatory. In this analysis, the main outcome parameter was modeled as a function of group assignment (binary covariate), the corresponding baseline value (linear covariate), and treatment expectation (linear covariate). Within this model, the adjusted group difference and its 95%-CI were estimated and tested for superiority of the intervention over the control group using a two-sided t-test at the *α* = 0.05 level. The analysis was consequently adjusted for baseline values and treatment expectation scores. Additionally, unadjusted means were reported to enhance transparency and enable comparison with the adjusted estimates. Secondary outcomes were analyzed using the same models as for the primary endpoint, but in an exploratory manner. Change scores and effect sizes (Cohen's d) were computed for between-group comparisons to assess the magnitude of the results. According to Cohen's thresholds, effect sizes were categorized as small (*d* = 0.2–0.49), moderate (*d* = 0.5–0.8), or large (*d* > 0.8) (36). All analyses were performed using R statistical software (version 4.0.2, R Foundation for Statistical Computing, Vienna, Austria). A *p*-value of less than 0.05 was considered to be statistically significant. All outcomes and analyses were specified in the registration. There are minor deviations from the registration: instead of using Hedges' g, as prescribed, we used Cohen's d. Furthermore, mediator analyses are only performed if the primary outcome is statistically significant. Consequently, no multiple mediator analyses were performed for secondary outcomes, as they are exploratory. No interim, subgroup, or sensitivity analyses were planned or conducted. Participants' free-text responses were reported descriptively in a narrative form.

## Results

### Study flow

A total of 135 participants were assessed for eligibility. Of those, 40 participants were included in the study and were randomly assigned to either the intervention group (*n* = 20) or the control group (*n* = 20). Enrollment and dropouts are shown in the CONSORT flow diagram (Figure 1). Thirty-one patients completed the intervention and the follow-up measurements. Six participants in the intervention group attended 50% or fewer of the sessions. However, they completed the questionnaires at both follow-up measurements and were thus included in the analysis. Overall, missing data across all questionnaire assessments amounted to 11.50%. Missing data rates were minimal at baseline (0.19%), increased to 14.2% at week 6, and were highest at week 26 (20.4%).

**Figure 1:**
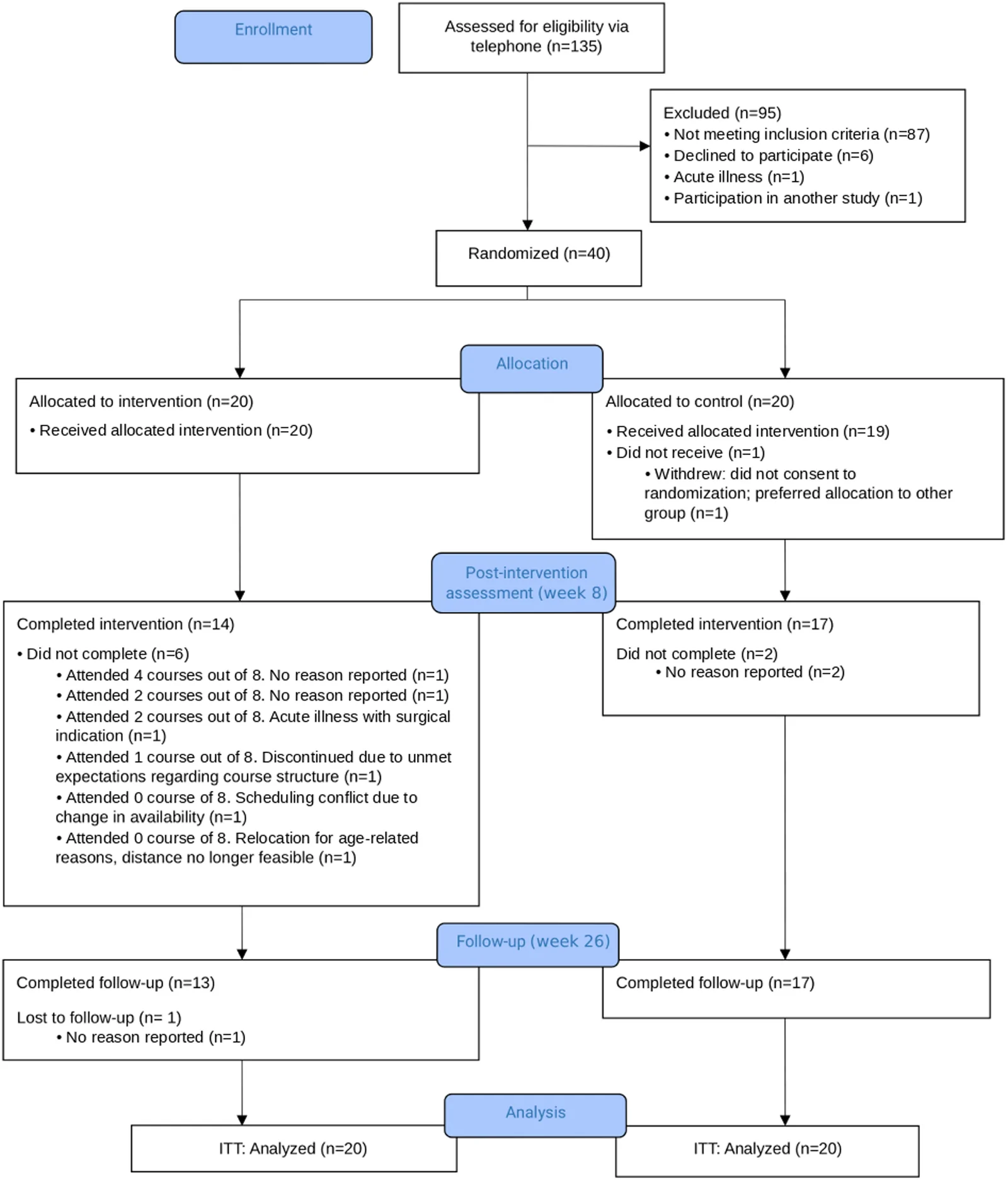
CONSORT flowchart.

### Demographic data

Table 1 shows the participants' baseline characteristics. Seventy-five percent of the participants were female, with a mean age was 61.8 years. Half of the participants were employed, while the other half were not in the labor force. The MBPM program group reported a longer duration of pain than the control group (16.8 years vs. 10.6 years). Seventy percent of the participants reported exercising at least once per week. Regarding active relaxation practice, most participants (55%) reported practicing active relaxation less than once per month. Only 27.5% reported practicing active relaxation one or two times per week.

**Table 1:** Characteristics of participants at baseline.

Variables (units)	MBPM program group (*n* = 20)		Waiting-list group receiving usual care (*n* = 20)		Total (*n* = 40)	
	mean ± SD		mean ± SD		mean ± SD	
Age *(years)*	61.6 ± 7.5		61.9 ± 9.8		61.8 ± 8.6	
Weight *(kg)*	77.4 ± 18.2		84.2 ± 15.4		80.9 ± 16.9	
BMI *(kg/m^2^)*	26.8 ± 5.8		28.7 ± 4.4		27.7 ± 5.3	
Pain duration *(years)*	16.8 ± 10.2		10.6 ± 8.4		13.9 ± 9.8	
Therapy expectation *(score)*	7.5 ± 1.7		7.2 ± 2.0		7.4 ± 1.8	
	*n*	%	*n*	%	*n*	%
Gender						
Female	17	85	13	65	30	75
Male	3	15	7	35	10	25
Marital status						
Partnered	6	30	4	20	10	25
Unpartnered	14	70	16	80	30	75
Cultural background						
Parents were born in Germany	12	60	18	90	30	75
At least one of the parents was not born in Germany	3	15	2	10	5	12.5
Not reported	5	25	0	0	5	12.5
Level of education						
Secondary or middle school	9	45	9	45	18	45
High school	7	35	4	20	11	27.5
University degree	4	20	7	35	11	27.5
Employment status						
Employed	10	50	10	50	20	50
Unemployed	0	0	0	0	0	0
Not in the labour force	10	50	10	50	20	50
Health insurance						
Statutory	16	80	10	50	26	65
Private or supplementary hospital insurance	4	20	10	50	14	35
Activity level						
Less than once per month	6	30	2	10	8	20
2–3x/Month	2	10	2	10	4	10
More than once per week	12	60	16	80	28	70
Active relaxation						
Less than once per month	10	50	13	65	23	57.5
2–3x/Month	3	15	3	15	6	15
More than once per week	7	35	4	20	11	27.5

BMI, body-mass index; kg, kilogram; m, meter; MBPM, Mindfulness-Based Pain Management; n, sample size; SD, standard deviation.

### Primary outcome

Figure 2 and Table 2 show the results of the MBPM program group and the waiting-list control group on the primary outcome at baseline, week 8 and week 26. By week eight, the MBPM program group had achieved a higher total CPAQ score than the waiting-list control group, with a moderate effect size (mean difference (MD) = 7.9; 95% CI [−0.4, 16.3]; *p* = 0.063; *d* = 0.6), though this difference was not statistically significant.

**Figure 2:**
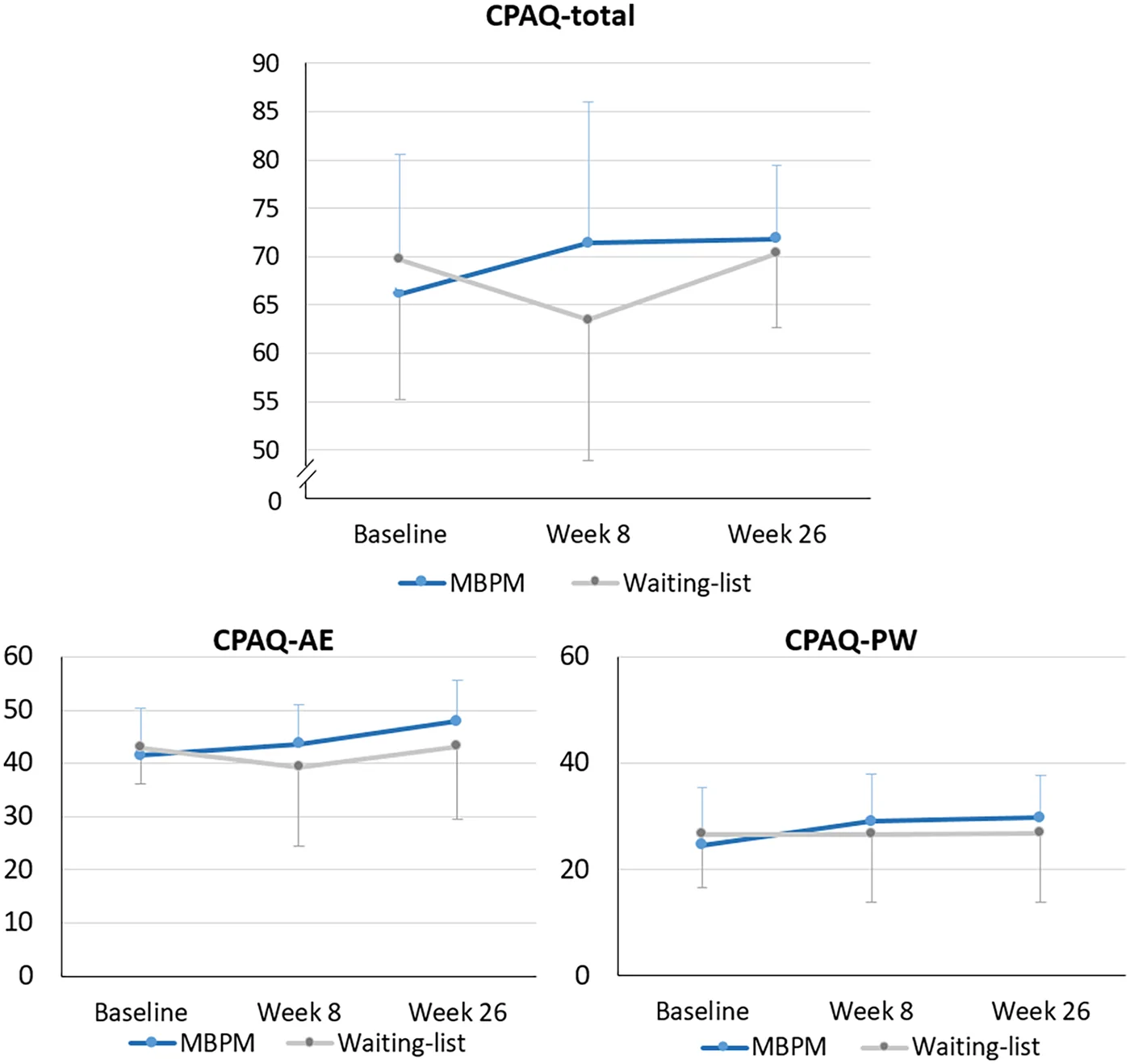
Line charts of the primary outcome at time points.

**Table 2:** Effects of the MBPM program on primary and secondary outcomes.

Outcomes		MBPM program group (*n* = 20) (mean ± SD)			Waiting-list group receiving usual care (*n* = 20) (mean ± SD)			Group differences^II^ week 8			Group differences^II^ week 26		
		Week 0	Week 8	Week 26	Week 0	Week 8	Week 26	Group difference (95% CI)	Cohen's d (95% CI)	*P*-value	Group difference (95% CI)	Cohen's d (95% CI)	*P*-value
CPAQ-total*	I	66.2 ± 15.9	70.1 ± 14.4	71.8 ± 6.5	69.7 ± 14.4	64.8 ± 19.6	70.3 ± 7.1	7.9 (−0.4; 16.3)	0.6 (−0.1; 1.3)	0.063	1.5 (−2.9; 6.0)	0.2 (−0.4; 0.9)	0.493
	II		71.4 ± 14.5	71.8 ± 7.8		63.5 ± 14.5	70.3 ± 7.7						
CPAQ-AE**	I	41.5 ± 8.8	43.1 ± 7.6	46.9 ± 7.1	43.0 ± 6.8	39.9 ± 14.1	43.6 ± 12.1	4.4 (−2.0; 10.7)	0.4 (−0.2; 1.1)	0.173	4.2 (−1.3; 9.8)	0.5 (−0.2; 1.1)	0.131
	II		43.7 ± 11.0	47.4 ± 9.6		39.3 ± 11.0	43.2 ± 9.6						
CPAQ-PW**	I	24.7 ± 10.9	28.3 ± 8.6	29.2 ± 7.3	26.7 ± 10.0	27.4 ± 12.2	27.6 ± 11.6	2.3 (−2.8; 7.4)	0.3 (−0.4; 0.9)	0.361	2.8 (−2.1; 7.7)	0.4 (−0.8; 1.0)	0.250
	II		29.0 ± 8.9	29.8 ± 8.5		26.7 ± 8.9	27.0 ± 8.5						
SF-12 physical**	I	33.0 ± 7.4	38.7 ± 9.1	41.7 ± 11.4	34.0 ± 9.9	39.4 ± 11.2	41.0 ± 12.8	−0.5 (−6.7; 5.7)	−0.1 (−0.7; 0.6)	0.873	0.5 (−6.6; 7.6)	0.0 (−0.6; 0.7)	0.884
	II		38.8 ± 10.8	41.6 ± 12.3		39.3 ± 10.8	41.1 ± 12.3						
SF-12 mental**	I	46.5 ± 12.7	51.6 ± 11.1	51.7 ± 10.2	50.6 ± 10.6	50.0 ± 12.4	50.0 ± 13.5	3.7 (−2.7; 10.2)	0.4 (−0.3; 1.0)	0.244	3.2 (−3.7; 10.1)	0.3 (−0.4; 1.0)	0.356
	II		52.7 ± 11.0	52.5 ± 11.9		48.9 ± 1.0	49.3 ± 11.7						
PDI**	I	25.6 ± 10.8	20.0 ± 10.7	18.8 ± 12.4	23.0 ± 14.5	22.5 ± 15.1	22.6 ± 15.097	−4.2 (−10.6; 2.2)	−0.4 (−1.1; 0.2)	0.187	−4.7 (−10.9; 1.5)	−0.5 (−1.2; 0.2)	0.130
	II		19.1 ± 11.1	18.3 ± 10.7		23.3 ± 11.1	23.0 ± 10.7						
HADS-A **	I	11.0 ± 2.2	11.5 ± 3.1	11.5 ± 2.3	11.5 ± 2.0	11.3 ± 1.8	11.3 ± 1.7	0.1 (−1.4; 1.7)	0.1 (−0.6; 0.7)	0.873	0.1 (−1.0; 1.2)	0.0 (−0.6; 0.7)	0.903
	II		11.4 ± 2.7	11.4 ± 1.9		11.3 ± 2.7	11.4 ± 1.9						
HADS-D**	I	9.1 ± 1.5	9.3 ± 1.9	9.4 ± 1.7	9.4 ± 1.5	10.1 ± 2.0	9.9 ± 1.8	−0.7 (−1.9; 0.5)	−0.4 (−1.0; 0.3)	0.232	−0.5 (−1.6; 0.7)	−0.3 (−0.9; 0.4)	0.410
	II		9.3 ± 2.1	9.4 ± 1.9		10.1 ± 2.1	9.9 ± 1.9						
ASES-D**	I	6.2 ± 1.8	6.8 1.5	7.0 ± 1.6	6.6 ± 1.6	6.8 ± 1.7	7.0 ± 1.9	0.2 (−0.8; 1.2)	0.1 (−0.5; 0.8)	0.651	0.2 (−0.7; 1.1)	0.2 (−0.5; 0.8)	0.637
	II		6.9 ± 1.7	7.1 ± 1.5		6.7 ± 1.7	6.9 ± 1.5						
CPSC**	I	1.8 ± 0.6	2.1 ± 0.6	2.1 ± 0.6	1.8 ± 0.6	2.0 ± 0.6	1.8 ± 0.6	0.2 (−0.2; 0.5)	0.3 (−0.3; 1.0)	0.344	0.3 (0.0; 0.7)	0.7 (0.1; 1.4)	**0**.**028**
	II		2.1 ± 0.6	2.1 ± 0.5		1.9 ± 0.6	1.8 ± 0.5						
SCS**	I	3.4 ± 0.6	3.7 ± 0.7	3.6 ± 0.7	3.3 ± 0.8	3.3 ± 0.7	3.4 ± 0.6	0.3 (0.0; 0.7)	0.7 (0.0; 1.4)	**0**.**035**	0.2 (−0.1; 0.4)	0.4 (−0.2; 1.1)	0.181
	II		3.6 ± 0.5	3.6 ± 0.5		3.3 ± 0.5	3.4 ± 0.5						
NRS**	I	6.2 ± 1.9	5.5 ± 1.7	4.9 ± 2.3	6.1 ± 1.6	5.5 ± 2.1	5.5 ± 2.2	0.0 (−1.0; 1.1)	0.0 (−0.6; 0.7)	0.934	−0.6 (−1.8; 0.6)	−0.3 (−1.0; 0.3)	0.348
	II		5.5 ± 1.8	4.9 ± 2.1		5.5 ± 1.8	5.5 ± 2.1						

**I:** unadjusted; **II:** adjusted for baseline measurement and treatment expectation.

* Primary outcomes.

** Secondary outcomes.

ASES-D, Arthritis Self-Efficacy Scale-Deutsch; CI, Confidence interval, CPAQ-AE/PW, Chronic Pain Acceptance Questionnaire-Activity Engagement/Pain Willingness; CPSC, Conscious Presence and Self Control; HADS-A/D, Hospital Anxiety and Depression Scale-Anxiety/Depression; MBPM, Mindfulness-Based Pain Management; NRS, Numerical Rating Scale for pain intensity; PDI, Pain Disability Index; SCS, Self-Compassion Scale; SD, standard deviation; SF-12, Short Form 12-Item Health Survey.

Bold values indicate statistical significance (*P* < 0.05).

### Secondary outcomes

Secondary outcomes are presented in Table 2 and Figure 2.

### Pain acceptance (CPAQ)

By week 26, the MBPM program group had higher total CPAQ scores than the control group (MD = 1.5; 95% CI [−2.9, 6.0]; *p* = 0.493; *d* = 0.2), though this difference was not statistically significant. Examining the CPAQ subscales revealed small to moderate effect sizes at weeks 8 and 26 for the CPAQ-AE and the CPAQ-PW subscale, though none were statistically significant.

### Health-related quality of life (SF-12)

There were no statistically significant differences between the groups on the physical subscale of the SF-12 at either week 8 (MD = −0.5; 95% CI [−6.7, 5.7]; *p* = 0.873; *d* = −0.1) or week 26 (MD = 0.5; 95% CI [−6.6, 7.6]; *p* = 0.884; *d* = 0.0). Similarly, no statistically significant differences were found in the mental subscale between the MBPM program group and the waiting-list group at week 8 (MD = 3.7; 95% CI [−2.7, 10.2]; *p* = 0.244; *d* = 0.4) and at week 26 (MD = 3.2; 95% CI [−3.7, 10.1]; *p* = 0.356; *d* = 0.3).

### Pain disability index (PDI)

The observed between-group differences at the PDI were not statistically significant at week 8 (MD = −4.2; 95% CI [−10.6, 2.2]; *p* = 0.187; *d* = −0.4) or at week 26 (MD = −4.7; 95% CI [−10.9, 1.5]; *p* = 0.130; *d* = −0.5).

### Hospital anxiety and depression scale (HADS)

For the HADS-A, the differences between the groups were small and not statistically significant at either time point (week 8: MD = −0.1; 95% CI [−1.4, 1.7]; *p* = 0.873; *d* = −0.1; and week 26: MD = 0.1; 95% CI = [−1.0, 1.2]; *p* = 0.903; *d* = 0.4). The HADS-D also revealed no statistically significant between-group differences (week 8: MD = −0.7; 95% CI [−1.9, 0.5]; *p* = 0.232; *d* = −0.4; week 26: MD = −0.5; 95% CI [−1.6, 0.7]; *p* = 0.410; *d* = −0.3).

### Arthritis self-efficacy (ASES-D)

Self-efficacy scores were not found to be statistically significantly different between the groups (week 8: MD = 0.2; 95% CI [−0.8, 1.2]; *p* = 0.651; *d* = 0.1; week 26: MD = 0.2; 95% CI [−0.7, 1.1]; *p* = 0.637; *d* = 0.2).

### Mindful awareness (CPSC)

No statistically significant group difference in mindful awareness wasdetected at week 8 (MD = 0.2; 95% CI [−0.2, 0.5]; *p* = 0.344; *d* = 0.3). At week 26, the MBPM program group had statistically significantly higher CPSC scores than the waiting-list group (MD = 0.3; 95% CI [0.0, 0.6]; p = 0.028; d = 0.7).

### Self-Compassion (SCS)

Statistically significant effects in favor of the MBPM program group compared to the control group were detected in terms of self-compassion by week 8 (MD = 0.3; 95% CI [0.0, 0.7]; *p* = 0.035; *d* = 0.7). However, by week 26, no statistically significant differences were observed (MD = 0.2; 95% CI [−0.1, 0.4]; *p* = 0.181; *d* = 0.4).

### Pain intensity (NRS)

The total pain intensity score did not differ significantly between the MBPM program group and the control group at week 8 (MD = 0.0; 95% CI [−1.0, 1.1]; *p* = 0.934; *d* = 0.0) and week 26 (MD = −0.6; 95% CI [−1.8, 0.6]; *p* = 0.348; *d* = −0.3).

### Participants' feedback

After the intervention, participants in the MBPM program indicated on the TCS that they would highly recommend the program. This sentiment was also reflected in the free-text sections of the questionnaires at weeks 8 and 26. Seven of the twenty participants in the intervention group provided feedback in these sections. The feedback was consistently positive. Many participants expressed gratitude for the support they received and for the opportunity to learn new techniques and gain new perspectives. Participants also expressed interest in using the intervention for similar complaints. Some participants expressed interest in continuing the program, which suggests that they found it acceptable.

### Adverse effects

No adverse effects (such as side effects or increased pain) were reported.

## Discussion

### Findings

This study evaluated an 8-week MBPM program based on the Breathworks method by Vidyamala Burch. The MBPM program was delivered in addition to usual care and was compared with usual care alone (waiting-list control). This study found no statistically significant difference in the primary outcome; pain acceptance between the MBPM program group and the control group at week 8. In terms of secondary outcomes, statistically significant differences between the groups were detected in self-compassion at the 8-week assessment, and in mindful awareness at the 26-week assessment. Both outcomes favored the MBPM program group. The other secondary outcomes revealed no statistically significant group differences.

### Comparison with prior research

In our study, pain acceptance was selected as the primary outcome. This choice is consistent with evidence from a recent meta-analysis that examined pain acceptance in MBIs for patients with chronic pain. The meta-analysis identified pain acceptance as a potential mechanism through which MBIs may promote engagement in daily activities despite ongoing pain (37). These findings support the idea that MBIs are not primarily intended to reduce pain intensity, but rather to modify pain perception by targeting emotional, cognitive, and psychological processes. Acceptance is one proposed process underlying these changes. However, pain acceptance did not show a statistically significant difference between the groups in our study. Furthermore, the confidence interval was wide, indicating substantial uncertainty in the estimated effect. Two systematic reviews have highlighted that the evidence regarding the effect of MBIs on pain acceptance is based on small and heterogeneous studies, making estimates imprecise and firm conclusions difficult (37, 38). Similarly, our study may have lacked the statistical power to detect smaller treatment effects, since the sample size was calculated based on an anticipated effect size larger than that observed.

A study by Pérez-Fernández investigated the effectiveness of the MBPM program in individuals with chronic pain. The results demonstrated greater improvements in the MBPM program group than the usual care group, with moderate-to-large effect sizes in terms of pain acceptance and management, psychopathological symptoms, and quality of life. However, participants were not truly randomized, as group allocation was based on the order of their arrival (21). The same research team performed an analysis of the long-term effects of the 8-week MBPM program. The results showed that the improvements were maintained at the 12-month follow-up, indicating positive long-term effects of the MBPM program for patients with chronic pain (39). Similarly, a study by Cusens et al. examining the MBPM program reported short- and long-term improvements concerning depression severity, and pain acceptance (22). The MBPM program was also associated with improvements in well-being, quality of life, and function without directly affecting pain intensity (22). However, limitations in allocation methods and comparator conditions restrict causal interpretation of these findings. Unlike the aforementioned studies, our study employed a randomized design, which may partly explain the more conservative findings.

Self-compassion showed a statistically significant short-term difference between groups, favoring the MBPM program group. A previous study on mindfulness- and acceptance-based interventions for chronic pain suggests that self-compassion can be improved by cultivating tolerance, kindness, and detachment toward personal experiences through mindfulness- and acceptance-based interventions (40). Another study examined the effectiveness of mindfulness-based stress management programs and found that these programs promote self-compassion and compassion toward others through various practices. Thus, participants scored higher than those in the control group, indicating less judgmental and friendlier attitudes toward themselves and others (41). In parallel, the findings of our study indicate that the MBPM program may be associated with more compassionate attitudes. This may be because the MBPM program directly targets compassion through practices such as kind awareness and loving kindness (21). However, these outcome comparisons do not establish a mechanism of change or greater responsiveness of self-compassion than other outcomes.

Self-compassion is particularly relevant in the context of chronic pain, where higher self-compassion has been associated with lower pain intensity, less catastrophizing, reduced anxiety and depression, and greater pain-related self-efficacy (42). Another study found that higher self-compassion was indirectly associated with diminished pain-related disability through greater self-efficacy (43). A systematic review by Greff Ballejos et al. suggested that self-compassion based interventions in adults with low back pain may offer potential benefits in terms of pain-related biopsychosocial parameters and mental health outcomes and reduce negative emotions, including pain-related stress and anger (44). Nevertheless, although self-compassion, pain acceptance, and mindfulness are potentially interrelated, they should be considered distinct concepts: mindfulness refers to nonjudgmental attention to the present moment (45), while self-compassion involves an emotional and motivational response characterized by compassion toward oneself when experiencing pain (46) and may motivate a number of adaptive behaviors (47). Interoceptive awareness may enable individuals to notice and interpret internal bodily signals without automatically perceiving them as threatening. This may provide a link between bodily experience and emotional regulation (48). Consistent with this proposed pathway, Lev Arey et al. found that even a single session of mindfulness self-compassion practice improved participants' interoceptive awareness and reduced their anxiety; however its effect on pain was less pronounced (49). These findings suggest that body awareness and emotional processes may respond differently to intervention than pain-related outcomes. Nevertheless, these findings do not definitively establish self-compassion as the underlying mechanism.

Pain acceptance requires both openness to pain and sustained engagement in activities (21) making it more behaviorally demanding more gradual process of change (50). This may help explain why pain acceptance showed a different short-term response in the present study. From this perspective, the statistically significant short-term difference in self-compassion favoring the MBPM program in our study should be considered an exploratory and hypothesis-generating; rather than evidence of clinical importance. Thus, the observed short-term between-group difference in self-compassion should be interpreted cautiously and warrants further examination in larger trials.

A previous study on the effects of an MBI among headache sufferers reported that having an accepting attitude toward changes in emotions, thoughts, and behavior patterns had a statistically significant effect on mindful awareness and mediated pain modulation (51). In our study, between group comparisons favored MBPM for mindful awareness at week 26; the difference at week 8 was not statistically significant. In contrast to our study, in a five-session mindfulness trial for chronic pain, mindfulness scores did not show a statistically significant change and the authors suggested considering a longer-term study or follow-up period to obtain more consistent results (52). In a previous study showed that mindfulness-related outcomes may depend on regular independent practice beyond the weekly sessions, as greater home practice has been associated with increases in mindfulness or facets of mindfulness facets in MBIs (53). Because home practice was not monitored in the present study, we cannot determine whether continued practice contributed to the week-26 finding.

Anxiety and depression are broader outcomes of emotional distress. In the most comparable MBPM trial, anxiety and depressive symptoms in individuals with chronic pain improved statistically significantly compared with the waiting-list control group (21). Two studies, including a pilot study of the MBPM program, reported reductions in psychological distress and improvements in depression and anxiety levels, possibly due to increased mental flexibility (22, 54). Conversely, a meta-analysis of mindfulness-based approaches concluded that these interventions may not directly affect anxiety and depression in patients with chronic pain, and improvements in psychological well-being were found to be inconsistent (55). Consistent with this meta-analysis, the present study found no statistically significant short- or long-term differences in anxiety and depression scores between the MBPM program group and the control group.

In the present study, no statistically significant differences in functional capacity or arthritis self-efficacy were observed between the groups. Consistent with our findings, a systematic review examining the effects of mindfulness skills training on physical function concluded that mindfulness-based approaches do not provide sufficient evidence to improve physical functioning (56). However, most of the RCTs included in the review focused on psychological variables related to pain. Furthermore, physical function was mostly assessed in limited detail, sample sizes were small, and different intervention responses may have emerged due to clinical heterogeneity.

### Strengths

This study has several key strengths. First, it is the first study examining the effects of the MBPM program on individuals with chronic musculoskeletal pain using a randomized controlled clinical trial design. Second, the randomized design and intention-to-treat analysis increase the study's internal validity and minimize potential selection bias. Third, the RCT was reported in accordance with the CONSORT statement to support a clear, transparent, and complete report. Fourth, using subjective, self-reported outcomes enables direct evaluation of perceived changes in pain-related parameters and well-being. Fifth, the use of ANCOVA allowed the estimate of the effects of the MBPM intervention more accurately by controlling for initial differences and treatment expectations. Lastly, the positive feedback from participants suggests that the MBPM program was acceptable to those who provided feedback.

### Limitations

Nevertheless, there are some limitations that should be acknowledged. The study included 20 participants in each group. for a total of 40 participants. This was based on a sample size calculation. However, the observed effect size estimate for the primary outcome (*d* = 0.6) was smaller than anticipated (*d* = 1.04). Thus, the calculated sample size may have been too small to detect smaller effects, suggesting that the intervention may be less potent than initially assumed or that the original estimate was overly optimistic. This overestimation could be due to differences in study populations, intervention fidelity, or methodological factorsTherefore, the findings should be interpreted with caution, recognizing that statistically non-significant results may reflect insufficient statistical power rather than a true absence of an effect. Additionally, testing multiple secondary outcomes without adjusting for multiple comparisons may have increased the risk of a type I error. Therefore, the statistically significant findings regarding self-compassion and mindful awareness should be interpreted cautiously and considered exploratory.

Seventy-five percent of our sample were female participants and the overall sample had a mean age of 61.8 years. This may limit the generalizability of the findings to broader and more diverse populations, such as younger individuals and men. Additionally, the sample included participants with various chronic musculoskeletal pain conditions. In addition, participants in the MBPM program group reported a longer pain duration at baseline than participants in the control group. This difference may reflect greater chronicity and reduced responsiveness to treatment. Furthermore, prior experience with mindfulness, meditation, or self-compassion was not evaluated and could have affected the response to the intervention. Additionally, the most common diagnosis was knee osteoarthritis (MBPM program: *n* = 8; control: *n* = 6), followed by neck pain (MBPM program: *n* = 3; control: *n* = 3). All other diagnoses, including fibromyalgia, osteoarthritis affecting the hands, feet, cervical spine and shoulders, lumbar disc herniation, lumbar spine syndrome, tension-type headaches, meniscal injuries, spinal canal stenosis, and lumbar osteochondrosis, were reported by no more than one participant as either a primary diagnosis or a coexisting condition. This variability in treatment response could have potentially diluted condition-specific effects. Therefore, the findings should be interpreted as reflecting the intervention's effects on a heterogeneous musculoskeletal pain population rather than on any specific diagnostic subgroup. While this improves the study's ecological validity, the variety of pain diagnoses may have obscured the specific effects of the MBPM program on different conditions, because these conditions may involve distinct underlying mechanisms, symptom profiles, and responses to the intervention.

Participants in both groups continued their usual care and could also start concomitant therapies during the study period. However, since changes in usual care and its therapies were not monitored, they should therefore be considered as a limitation. It cannot be ruled out that changes in concurrent treatments influenced the observed outcomes. Nevertheless, providing the intervention alongside usual care may enhance external validity because the complementary nature of the MBPM program reflects real-world conditions. The MBPM program was evaluated in comparison with a waiting-list control group. As this comparator did not control for nonspecific factors such as group support, therapist attention, therapy expectations, and structured participation, the specific effects of the MBPM program cannot be clearly identified. Further, as often is the case in studies involving non-pharmacological interventions, it was also not possible to blind the therapists and participants due to the nature of the procedure. This may have led to bias regarding expectations or responses. Therefore, treatment expectations were assessed at baseline using the TCS, and the analyses were adjusted accordingly. Consequently, potential biases related to expectations may have been reduced through statistical control.

Another limitation was the lack of detailed data on intervention attendance and home practice engagement, which limited the ability to assess adherence. The absence of adherence data posed a significant challenge in determining whether the lack of statistically significant effects on pain acceptance, awareness, or pain intensity was due to limited intervention efficacy, insufficient practice, variable participant engagement, or inadequate measurement sensitivity. Additionally, intervention fidelity is an important factor that was also not formally assessed in our study. Further, missing data increased over time, and the estimates, particularly for secondary outcomes at week 26, may have been affected. MissForest imputation was used, but no complete-case sensitivity analyses were conducted. Therefore, the robustness of the imputed results should therefore be interpreted with caution.

### Implications for future trials and clinical practice

Although no intervention-related adverse events were reported, there is currently insufficient evidence from this trial to support the clinical use of the MBPM program. These findings underscore the need for further more rigorously designed studies.

For future studies, sample size calculations should use the more conservative observed effect size (*d* ≈ 0.5–0.6) rather than the initial estimate. This adjustment will likely require substantially larger sample sizes to achieve adequate statistical power (e.g., 80%–90%). Additionally, researchers should consider strategies to enhance statistical power beyond increasing sample size alone, such as improving intervention adherence, recording intervention fidelity, and reducing outcome variability by establishing more refined inclusion criteria. Incorporating pilot data and conducting sensitivity analyses during the planning phase can further support more accurate and realistic power estimations, ultimately yielding more robust and reliable findings.

To improve external validity, future studies should aim to recruit more diverse samples, including younger individuals and a greater number of male participants. They should also examine whether the effects of the MBPM program differ according to specific pain diagnoses and relevant characteristics, such as pain duration, psychological distress at baseline, prior mindfulness experience, and self-compassion at baseline. Additionally, follow-up periods longer than 26 weeks are necessary to evaluate the long-term sustainability of treatment effects and the potential recurrence of symptoms. Future research should examine whether periodic booster sessions or other maintenance strategies could improve the intervention's long-term effectiveness. Future studies should also include monitoring of other treatments used during the study period, intervention attendance, fidelity, and home practice. Finally, using structurally similar active control conditions instead of waiting-list controls is recommended to more clearly identify the specific effects of the MBPM program.

In terms of participant attrition, in the present trial it was primarily attributed to factors including reluctance to accept randomization, with a preference for allocation to a specific group, scheduling conflicts due to changes in personal availability, and unmet expectations regarding the structure of the intervention. These findings highlight both methodological and practical barriers to sustained engagement. To enhance participation in future studies, several strategies are recommended. First, improving participants' understanding and acceptance of randomization through clear communication may reduce resistance. Second, incorporating alternative designs such as preference-based approaches, may also help. Additionally, increasing flexibility in scheduling, such as offering multiple session times, and hybrid or online formats, can help accommodate varying schedule es. Finally, providing detailed and realistic pre-intervention information, including orientation sessions or sample content, can better align participants' expectations with the program's actual structure, thereby reducing dropout rates and improving overall retention.

## Conclusion

The study found that the MBPM program did not have a statistically significant effect on the primary outcome pain acceptance in adults with chronic specific or nonspecific musculoskeletal pain compared to the waiting-list control group at the 8-week assessment. However, the effect size for the primary outcome was moderate and favored the MBPM program. No statistically significant differences were observed for the secondary outcomes, except for self-compassion and mindful awareness at different follow-up points, which favored the MBPM program. However, these findings should be regarded as hypothesis-generating, and their clinical importance remains uncertain. No intervention-related adverse events were observed. These findings underscore the need for larger, more rigorously designed with longer follow-up periods studies to further evaluate the effectiveness of MBPM for individuals with chronic pain.

## Data Availability

The raw data supporting the conclusions of this article will be made available by the authors, without undue reservation.
